# *Bacillus velezensis* strain B26 modulates the inflorescence and root architecture of *Brachypodium distachyon* via hormone homeostasis

**DOI:** 10.1038/s41598-022-12026-6

**Published:** 2022-05-13

**Authors:** Meha Sharma, Jean-Benoit Charron, Mamta Rani, Suha Jabaji

**Affiliations:** Department of Plant Science, Macdonald Campus of McGill University, 21,111 Lakeshore Rd., Ste-Anne de Bellevue, QC H9X 3V9 Canada

**Keywords:** Microbiology, Molecular biology, Plant sciences

## Abstract

Plant growth-promoting rhizobacteria (PGPR) influence plant health. However, the genotypic variations in host organisms affect their response to PGPR. To understand the genotypic effect, we screened four diverse *B. distachyon* genotypes at varying growth stages for their ability to be colonized by *B. velezensis* strain B26*.* We reasoned that B26 may have an impact on the phenological growth stages of *B. distachyon* genotypes. Phenotypic data suggested the role of B26 in increasing the number of awns and root weight in wild type genotypes and overexpressing transgenic lines. Thus, we characterized the expression patterns of flowering pathway genes in inoculated plants and found that strain B26 modulates the transcript abundance of flowering genes. An increased root volume of inoculated plants was estimated by CT-scanning which suggests the role of B26 in altering the root architecture. B26 also modulated plant hormone homeostasis. A differential response was observed in the transcript abundance of auxin and gibberellins biosynthesis genes in inoculated roots. Our results reveal that *B. distachyon* plant genotype is an essential determinant of whether a PGPR provides benefit or harm to the host and shed new insight into the involvement of *B. velezensis* in the expression of flowering genes.

## Introduction

*Bacillus* species are one type of rhizobacteria that can boost plant growth through the induction of antibiosis, facilitating nutrient availability through the synthesis of phytohormones, and competitive omission^1^. Such interactions help in endurance and adaptation of both host and PGPR in any stress environment^2^. We previously demonstrated that *Bacillus velezensis* strain B26, is a growth-promoting bacterium of timothy grass and the model plant *Brachypodium distachyon*, which enhanced the growth and accelerated flowering time through the production of hormones, volatiles and various antimicrobial compounds^3,4^. We also showed that strain B26 improves the growth of these grasses under extended drought conditions by modulating the expression of drought-responsive genes in *B. distachyon,* and also by the modification of osmolytes in roots and shoots of timothy grass^3^. Successful colonization of *B. distachyon* roots by strain B26 is based on the composition of roots exudates (the type of organic acid and their biosynthetic genes), chemotaxis and the induction of biofilm and their encoding genes^5^.

It is well established that plant genotype can impact the degree of plant growth-promotion of some PGPR^6^. The effects of inoculation of 20 rice cultivars of genetically distinct groups with *Azospirillum* sp. provided varied results in terms of the number of tillers^7^. Also, different accessions of *Arabidopsis* displayed different microbial communities, indicating that plant host genetic factors shape the associated microbiota^6,8^. The genotypes of the model grass *B. distachyon* has an important role in defining the plant host responses to PGPR^9^. However, it is unclear whether the host’s genotypic variations affect the microbiome in such a way that leads to adaptive consequences to the host. The study of Do Amaral et al*.*^9^ and others only described the short-term growth responses on plants^10^.

*B. distachyon* is closely related to cultivated monocotyledons such as rice, wheat, and maize, and is a model plant to study plant–microbe interactions and stress tolerance^4,11,12^. Due to ease in genetic transformation, *B.distachyon* is ideal for generating transgenic lines^13^. Various transgenic lines have been generated in the background of *B.distachyon* accession line Bd21-3 with loss and gain of function of a target gene^14,15^. Moreover, *B. distachyon* accessions exhibit variation in various phenotypic traits^16^.

The reproductive success of many plants hinges on flowering^17^. Flowering responds to environmental cues such as long exposure to cold temperatures (i.e., vernalization) and photoperiods (i.e., variation in day length). The regulation of the flowering process in *B. distachyon* is controlled by several key genes, which include *VERNALIZATION 1* (*VRN1*), *VRN2* and *FLOWERING LOCUS FT1* (*FT1*)^17–19^.The expression of these genes is affected by temperature and photoperiods^20^. It was demonstrated that the over-expression of *FT1* accelerates flowering in *B. distachyon* and wheat^17,21^. However, the flowering pathways are not limited to the shoot apical meristem where flowers are originated, but it depends on shoot–root communication^22,23^. For example, the majority of flowering genes in *Arabidopsis* and *Cassava* are variably expressed when plants are exposed to photoperiod that induces flowering^22,23^.These studies provide a new understanding on the involvement of the root in the flowering process. Signalling molecules from roots including phytohormones modulate shoot growth and root architecture^24^. Additionally, the plant growth stimulation by beneficial rhizobacteria has been associated with the biosynthesis of plant growth regulators produced by rhizobacteria including auxins, gibberellins, cytokinins and ABA^25^. These microbial signals alter the plant hormone levels. Previously, we reported on the beneficial traits mediated by phytohormones produced by *B. velezensis *strain B26^4^ causing increased fitness of plant resulting in 121% more spikelets in inoculated *B. distachyon* than the respective control^3^. Despite significant advances in plant-rhizobacteria interactions, regulation of plant flowering genes in response to rhizobacteria is scarce^26^.

Here, we aim to (i) study the potential use of *B. distachyon* genotypes for studies of PGPR-grass interactions throughout the whole growth cycle of the genotypes. (ii) characterize the responses of expression patterns of selected flowering genes to *B. velezensis* inoculation in *Brachypodium* wild accessions and (iii) understand whether strain B26 could alter the expression of *Brachypodium* transgenic lines overexpressing flowering genes relative to the colonized wild type (iv) understand whether growth promotion by strain B26 is differentially associated with phytohormone homoeostasis and transcript abundance. We screened four diverse genotypes of *Brachypodium* for their ability to be colonized by *B.velezensis.* We reasoned that *B. velezensis* may have an impact on the inflorescence and root architecture of *B. distachyon* genotypes.

## Results

### Bacterial inoculation elicited varied growth response of *B. distachyon* accessions

**Figure 1:**
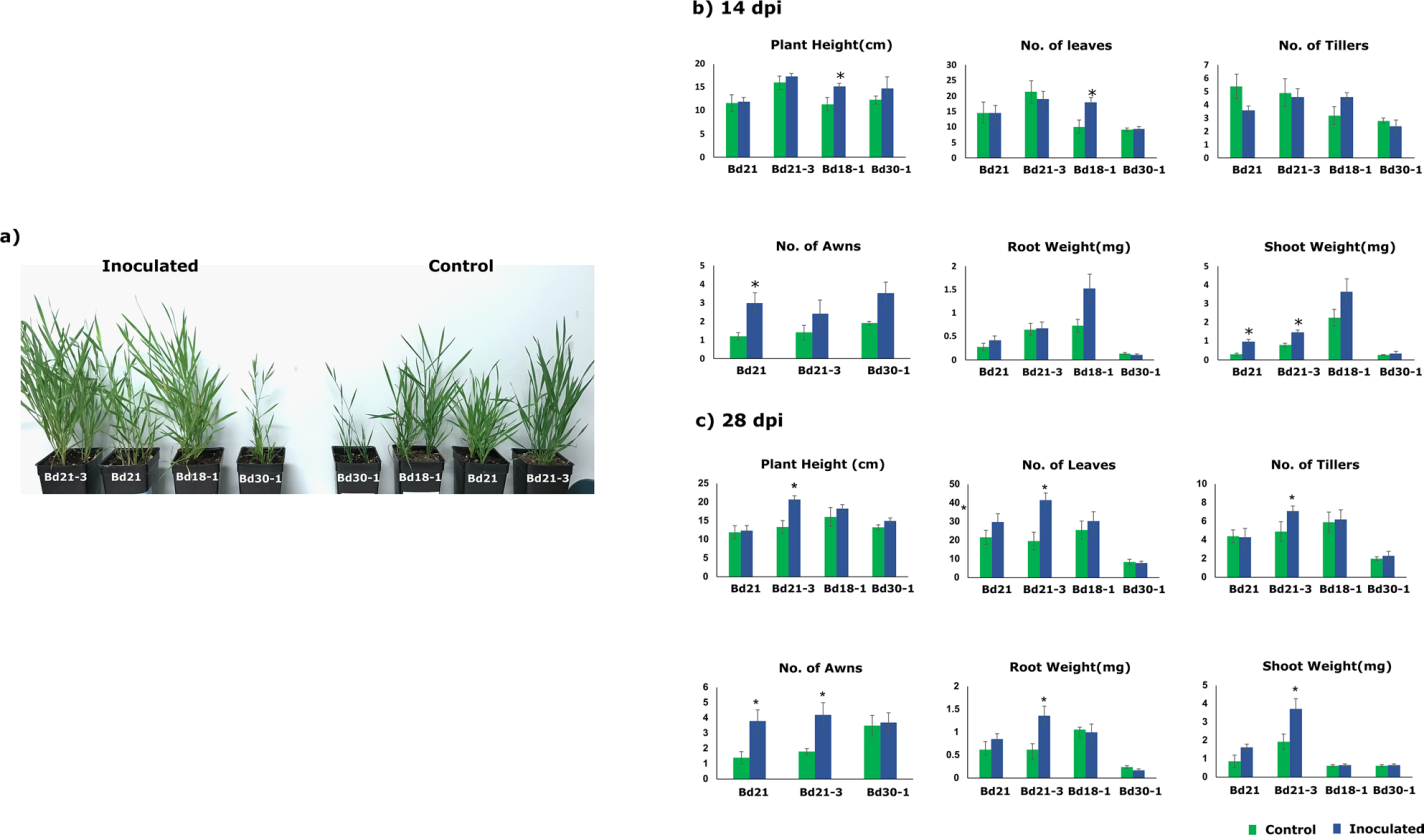
(**a**) *Brachypodium* accession lines displaying growth response at 28 days post-inoculation (dpi). The left panel of (**a**) shows accession lines (Bd21-3, Bd21, Bd18-1 and Bd30-1) inoculated with *Bacillus velezensis* strain B26. The right panel shows control accession lines (**b**) Growth response parameters (Plant height, No. of leaves, No. of tillers, No, of awns, Root weight and shoot weight) of wild type *B.distachyon* genotypes in response to B26 inoculation at 14 days post-inoculation (dpi) (**c**) at 28 dpi. Bars represent the mean of five biological replicates. t-test was used to determine statistical differences between inoculated and non-inoculated plants. * indicates significance according to Independent Student t-test (*p* < 0.05). Note: Bd18-1 did not flower at 14 and 28dpi.

A differential response was observed in Bd21, Bd21-3, Bd18-1 and Bd30-1 in response to B26 colonization (Fig. 1a). At 14 days post inoculation (dpi), a significant increase of 150% in the number of awns and 250% increase in the shoot weight of inoculated accession Bd21 compared to non-inoculated control was observed (Fig. 1b, Table S2). The plant height and number of leaves of inoculated Bd18-1 increased by 34% and 78%, respectively compared to the control. At 28 dpi, Bd21-3 showed a significant increase in all growth parameters compared to the control (Fig. 1c). While Bd 30-1 at 28dpi, did not show a significant response to B26 inoculation as indicated by the growth parameters (Fig. 1c). However, there was no difference in flowering time of inoculated and non-inoculated plants. Control and inoculated accessions flowered at the same time but an increase in the number of awns was observed.

### *B. distachyon* accessions sustained populations of strain B26 in root and shoot tissues

Quantification was done in roots and shoots of *B. distachyon* accession Bd21-3 that responded well to B26 inoculation in terms of growth parameters, and accession Bd30-1 that showed similar growth responses to B26 as the control after 14 dpi and 28 dpi (Table 1). Strain B26 had similar sustaining endophytic populations in roots and shoots in both genotypes. In the case of Bd21-3, more copies were found in roots at 28dpi as compared to shoots (Table 1). On the contrary, Bd30-1 had more copies in shoots at 14dpi. However, more B26 gene copies were found in tissues of Bd21-3 as compared to Bd30-1.

**Table 1:** Determined Cq values and gene copy number of B26 leaf and root samples of Bd21-3 and Bd30-1 inoculated with B26.

Accession	dpi	Average Cq^$^ values		Gene copies/g of sample		Log_10_	
		Shoot	Root	Shoot	Root	Shoot	Root
Bd21-3	14dpi	32.8 ± 0.6^b^	32.8 ± 0.6^b^	855,754	1,003,888	5.9	5.9
	28dpi	32.7 ± 0.5^b^	30.2 ± 1.2^a^	903,143	5,227,176	5.9	6.7
Bd30-1	14dpi	32.5 ± 0.8^b^	34.8 ± 1.2^a^	1,124,891	274,849	6	5.3
	28dpi	33.7 ± 1.1^b^	34.9 ± 1.4^a^	539,400	259,528	5.7	5.3
	42dpi	33.3 ± 0.7^b^	34.9 ± 0.9^a^	631,073	238,371	5.8	5.3

^$^Cq values -quantification cycle (Cq).

^a,b^Letter to represent the significant difference. Means with same letters are not significant, while means with different letters are significant within column according to Independent Student t-Test (*p* < 0.05).

### Differential expression patterns of selected flowering genes in inoculated *B.distachyon* genotypes

**Figure 2:**
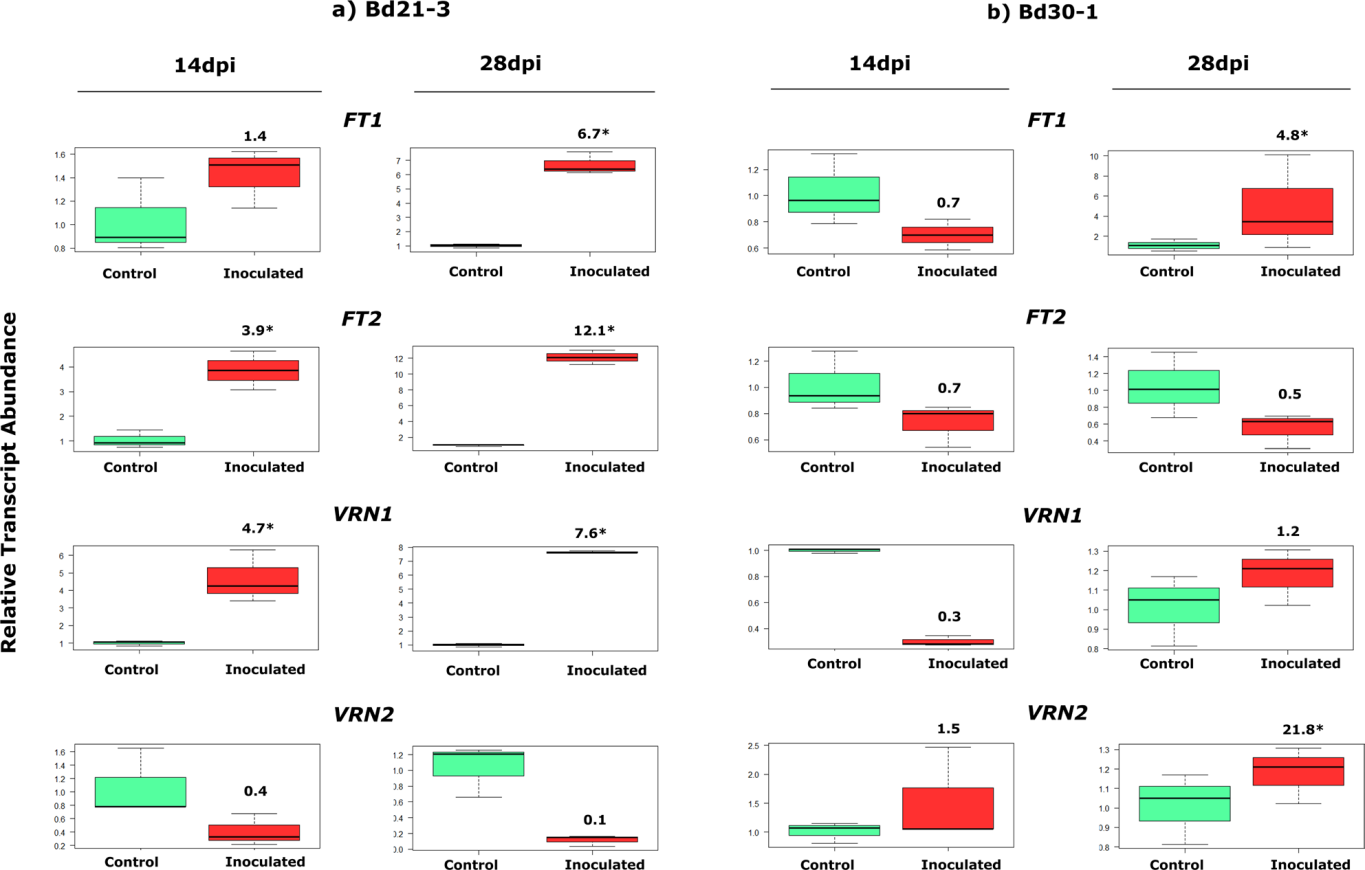
A comparison of relative transcript abundance of flowering genes (*FT1, FT2, VRN1* and *VRN2*) in shoots of control and inoculated (**a**) Bd21-3 (rapid flowering line) and (**b**) Bd30-1(intermediate flowering line) at 14 and 28dpi. Numbers above the box plot represent fold change. * indicates significance according to Independent Student t-test (*p* < 0.05).

The expression analysis of flowering genes: *FT1, FT2, VRN1* and *VRN2* in leaves in response to B26 inoculation of Bd21-3 and Bd30-1 is depicted in Fig. 2. Significantly higher expression levels (*p* < 0.05) of *FT1* (6.70-fold); *FT2* (12.1-fold); and *VRN1* (7.6-fold) transcripts were detected in inoculated Bd21-3 compared to the control at 28 dpi (Fig. 2a). The expression of *VRN2* in response to B26 was similar to the control. Inoculation of B26 in genotype Bd30-1, showed a significant up-regulation in *FT1* transcript abundance (4.8-fold) at 28dpi. (Fig. 2b). In contrast, to Bd21-3, a substantial increase (21.8-fold) in *VRN2* transcript levels in inoculated Bd30-1 was detected at 28 dpi.

### Strain B26 improves root and shoot weights of transgenic lines

**Figure 3:**
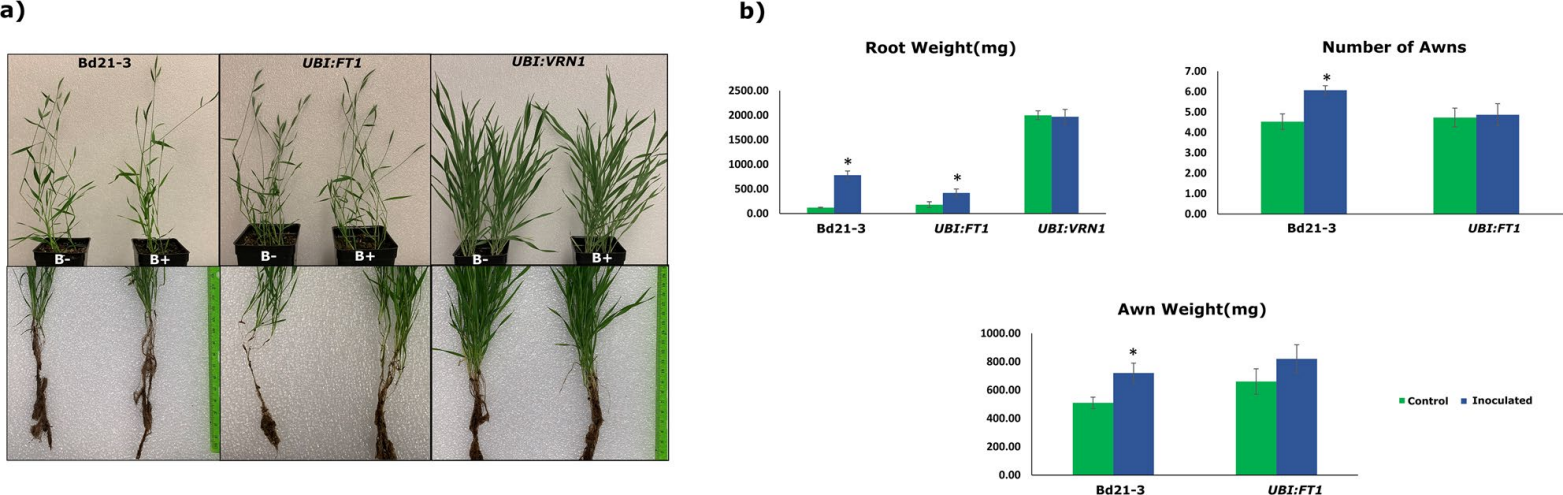
(**a**) Comparison of shoot (upper panel) and root (lower panel) phenotypes at 28 dpi between inoculated (B+) *Brachypodium* wild type Bd21-3 and transgenic lines *UBI:FT1*, *UBI:VRN1* and their respective controls (B−). (**b**) Comparison of growth parameters of inoculated Bd21-3 and transgenic lines with non-inoculated control plants. Standard errors are displayed for each bar graph. Independent Student t-test was used to determine statistical differences. *indicates significance (*p* < 0.05). Each bar represents the mean of 5 replicates.

Detection of transgene in *UBI:FT1* and *UBI:VRN1* was done by PCR. cDNA specific forward primer and pANIC vector AcV5 tag reverse primer were used to detect transgene in transgenic lines. PCR with *VRN1-F* /*FT1-F* and AcV5 tag yielded an expected band size of approximately 260 bp and 500 bp, respectively which confirmed the presence of transgene (Fig. S1a,b). No amplification was observed in wild type Bd21-3 as there is no transgene present. A wide differential growth response among the transgenic lines compared to the wild type genotype Bd21-3 was observed (Fig. 3a). At 28 dpi, the root and shoot weights of transgenic line *UBI:FT1* significantly increased by 132% and 162%, respectively in response B26 (Table S3). Growth parameters such as the number of awns, root and awn weight of the wild type genotype Bd21-3 increased significantly by 34%, 52% and 43%, respectively (Fig. 3b, Table S3). No significant difference was observed between inoculated and control *UBI:FT1* at 14 dpi except for root weight.

### Strain B26 modifies root volume of wild type and transgenic lines

B26 inoculation had a positive effect on the root volumes as estimated by macro CT-scanning. An increase of 3.56, 1.67 and 1.90 times, respectively in the root volume of wild type Bd21-3, transgenic lines *UBI:FT1* and *UBI:VRN1* inoculated roots as compared to control roots (Table 2).

**Table 2:** Estimated root volume of *B.distachyon* wild type and transgenic lines from CT-Scanning data.

Accessions	Control (mm^3^)	Inoculated (mm^3^)	Difference
Bd21-3	425.7	1517.5	1091.8
*UBI:FT1*	881.2	1480.2	599
*UBI:VRN1*	637	1213	576

### Transcript abundance of flowering genes in roots and leaves of inoculated transgenic lines

**Figure 4:**
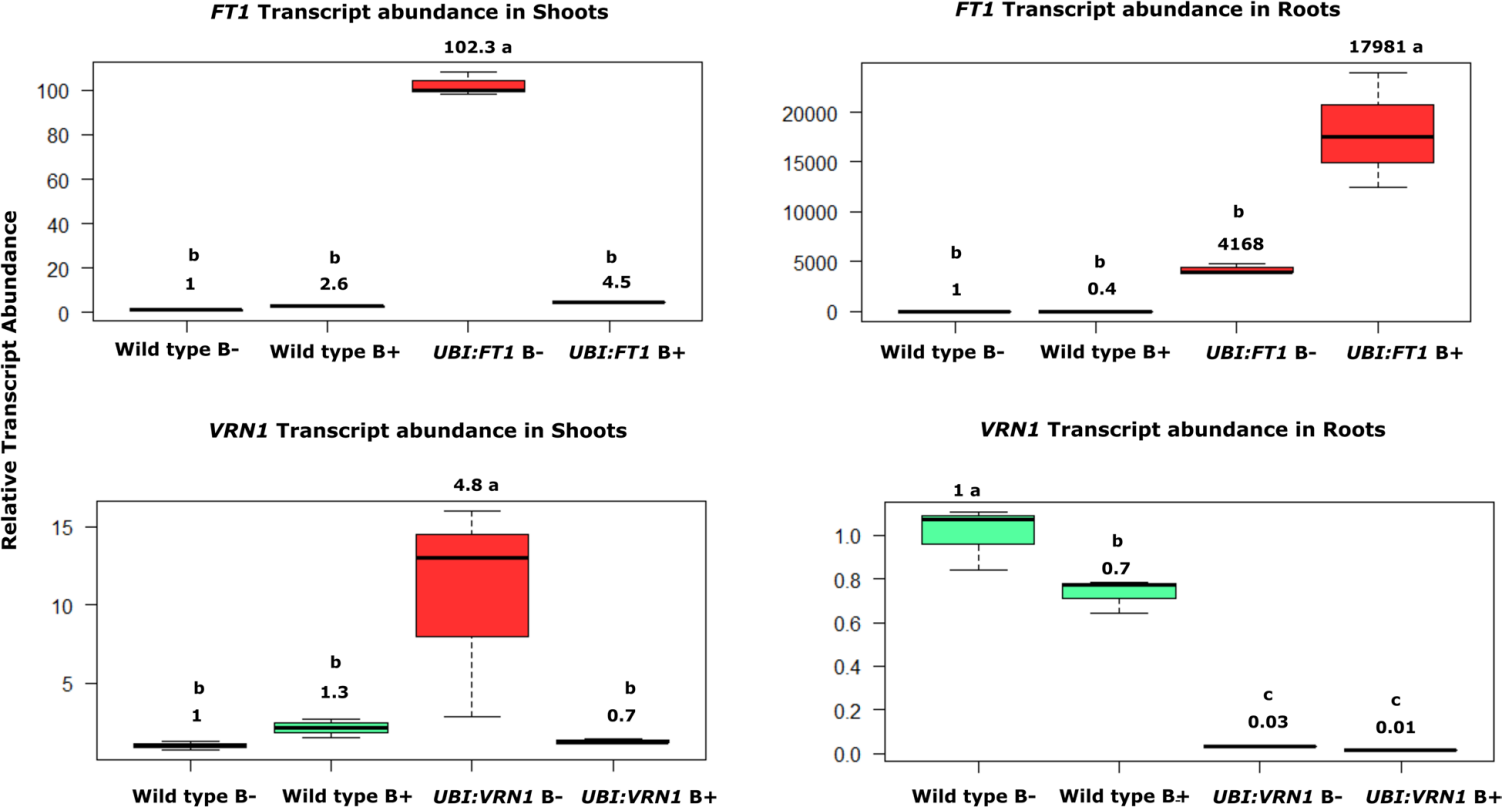
Comparison of relative transcript abundance of flowering genes in shoots and roots of inoculated (B+) and control (B−) wild type Bd21-3 and transgenic lines *UBI:FT1*, *UBI:VRN1* at 28 dpi. Numbers above the box plots represent fold change. Different alphabet above each box represent significance according to Tukey’s test (*p* < 0.05).

At 28 dpi, the phenotypic observations of flowering transgenic lines (Fig. 3a, b) showed the effect of inoculation is more noticeable in roots and awns of transgenics. This prompted us to study the expression of flowering genes in both roots and shoots of transgenic lines at this growth stage. Each transgenic line was compared with the wild type separately. A significant upregulation in transcripts of *FT1* gene (17,981-fold) was observed in inoculated roots of *UBI:FT1* relative to non-inoculated wild type. Strain B26 did not induce *FT1* nor *VRN1* genes in shoot tissues of inoculated transgenic plants. However, transcripts levels of *VRN1* gene were down-regulated in both roots and shoots of *UBI:FT1* and *UBI:VRN1* compared to the inoculated wild type (Fig. 4).

### B26 affects phytohormone homeostasis

#### Quantification of the endogenous level of phytohormones

**Figure 5:**
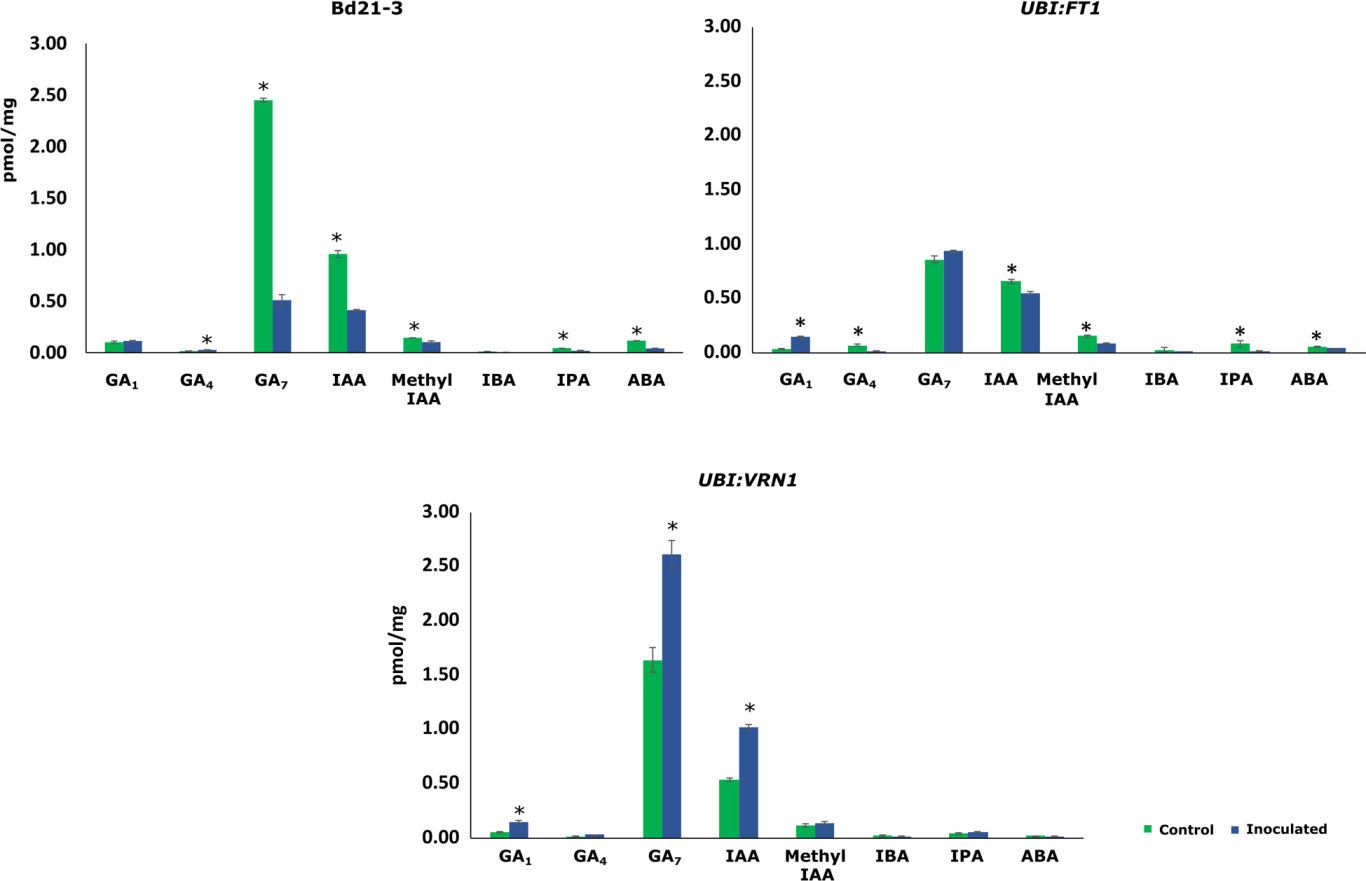
Quantification of phytohormones concentrations (pmol/mg) in inoculated (B +) and control (B−) of wild type Bd21-3 and transgenic lines *UBI:FT1*, *UBI:VRN1*. Bars represent the mean of five biological replicates. GA, gibberellic acid (GA_1_,GA_4_ GA_7_), IAA, (indoleacetic acid), IBA (indole butyric acid) and IPA (indole-3-propionic acid).

To complement earlier observations of the growth promotion of inoculated wild type Bd21-3 and transgenic lines, we measured the endogenous levels of phytohormones. Indole acetic acid (IAA), indole butyric acid (IBA) and indole -3-propionic acid (IPA), abscisic acid (ABA), kinetin and zeatin(cytokinin), gibberellins A_1_, A_3_, A_4_ and A_7_, were measured in roots of control and inoculated plants. Irrespective of the treatment, gibberellins (GA) were the most abundantly detected phytohormones. The phytohormone homeostasis in Bd21-3 was significantly affected by B26. Growth promotion of the wild type Bd21-3 by strain B26 is significantly (*p* < 0.05) associated with increases in GA_4_ (2-fold). While the amount of GA_7_, and IAA were significantly less by 4.8 and 2.3-fold ,respectively as compared to control roots (Fig. 5). In case of *UBI:FT1*, GA_1_ was significantly higher in inoculated roots than control. However, the concentration of other phytohormones was detected less in inoculated *UBI:FT1* roots as compared to control roots. In contrast, levels of GA_1_, GA_7_ and IAA were 2.75, 1.59 and 1.89 times respectively higher significantly in inoculated roots of *UBI:VRN1* when compared to control roots. However, Kinetin, Zeatin and GA_3_ were below the detection level.

#### Transcript abundance of genes related to phytohormones in Bd21-3

**Figure 6:**
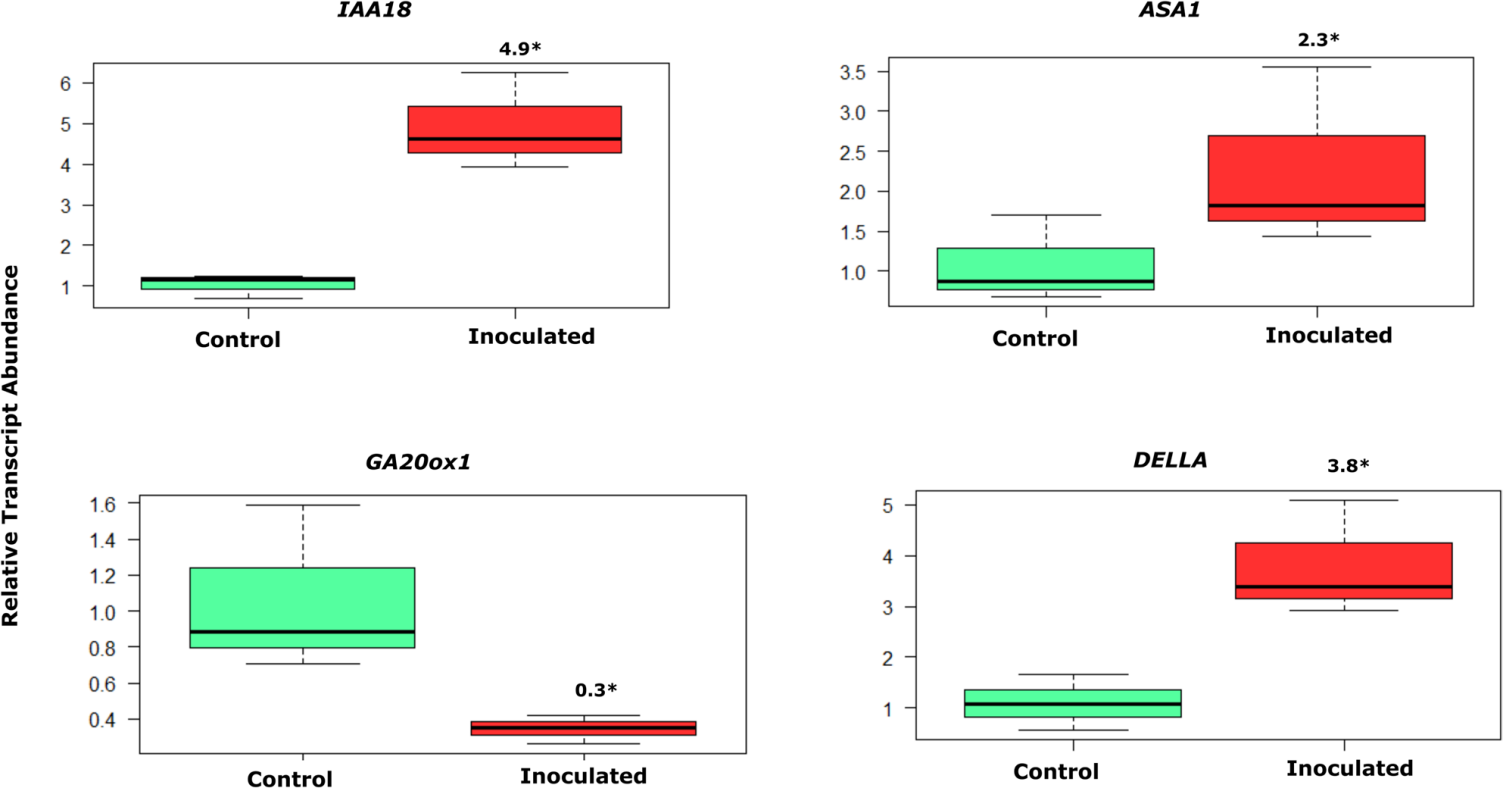
Relative transcript abundance of genes encoding biosynthesis of auxin and gibberellins in roots of control and inoculated Bd21-3 at 28 dpi. Numbers above the box plots represent fold change. Independent Student t-test was used to determine the statistical differences between inoculated and control roots. *indicates significance (*p* < 0.05).

A significant upregulation in transcripts of genes related to auxin biosynthesis was observed in wild type Bd21-3 only. *Anthranilate synthase alpha subunit 1(ASA1)* which catalyses the rate-limiting step of tryptophan biosynthesis^27^ and Indole-3-acetic acid inducing gene (*IAA18)* were significantly up-regulated by 2.3 and 4.9-fold, respectively in inoculated roots as compared to control roots (Fig. 6). A significant downregulation was observed in transcript abundance of *GA20ox1* which encodes gibberellin 20-oxidase enzyme that is involved in the later steps of the gibberellins (GA) biosynthesis pathway^28^ Interestingly, DELLA proteins, a key negative regulator of GA signalling^29^ was significantly up-regulated by 3.8-fold in inoculated roots as compared to control roots (Fig. 6).

## Discussion

The data presented here indicate that *B. distachyon* is a useful model to study PGPR-plant association and could serve as a model for rice and wheat. A central finding in this study is that plant genotype is a crucial determinant of whether rhizobacteria inoculation promotes plant growth or not. The four genotypes behaved differently throughout the whole life cycle of the plants for each growth parameter and showed statistically positive or negative responses for one or more of the parameters tested. Such response is exemplified in genotype Bd21 and Bd21-3 in which induction of flowering was accelerated in response to B26. These results are not uncommon among plant accessions since naturally occurring resistance is common in studies of plant–microbe interactions. *B. distachyon* genotypes demonstrated significant and varied responses to infection by pathogenic insects and fungi^30^. Moreover, several *B. distachyon* genotypes differed in their ability to associate with two diazotrophic strains and several genotypes responded negatively to the strains^9^. Also, wild accessions of *Arabidopsis thaliana* showed reduced growth in response to *Pseudomonas fluorescence*^8^. Of interest, genotype Bd30-1 which performed less favourably among the other 3 accessions, had sustained B26 populations in roots and shoots, but was insufficient to induce growth promotion in accession Bd30-1. This suggests that a different mechanism is implicated, and this requires further analysis.

Molecular studies on the regulation of flowering genes (*FT1*, *FT2, VRN1* and *VRN2)* in response to environmental cues have been intensively studied in *Arabidopsi*s, cereals^31,32^, and *B. distachyon*^17^. However, molecular studies on the regulation of flowering genes in response to rhizobacteria are scarce^26,33^. Flowering in *B. distachyon* is mostly regulated by three key genes viz., *VERNALIZATION1* (*VRN1*), *VRN2*, and *FLOWERING LOCUS T* (*FT*). *VRN1*, *VRN2* and *FT* form a regulatory loop in wheat and barley^34–36^. We focused on studying transcript levels of flowering genes in Bd21-3 a genotype known as rapid flowering and Bd30-1 a genotype known to show intermediate flowering. The inoculation of genotype Bd21-3 with strain B26, induced an abundance of *FT1* transcript levels in shoots and it was not a limiting factor in the upregulation of *VRN1.* Our results are in agreement with the elevated expression patterns of *FT1* and *VRN*1 in the rapid flowering *B. distachyon* accessions^17^. Intriguingly, this trend supports the proposed model for wheat and barley during cold exposure^37,38^. However, *VRN2* acts as a repressor of flowering and was expressed at lower levels in spring accession of wheat and barley^19^. In *B. distachyon, VRN2* was also expressed at lower levels in the spring accession Bd21-3^20^. The current study supports this evidence since *VRN2* was down-regulated in Bd21-3 accession line. In the case of the intermediate flowering accession line, Bd30-1, the expression of *VRN2* was remarkably high compared to Bd21-3. Similar results were obtained by Ream et al*.*^17^ in which Bd2-3 had more amounts of *BdVRN2* and less amount of *BdFT1*, suggesting that *VRN2* may play a role as a flowering repressor. Both Bd2-3 and Bd30-1 belongs to the Intermediate rapid flowering class.

To fully understand the role of B26 inoculation on flowering genes, we tested overexpressing flowering transgenic lines *UBI:FT1* and *UBI:VRN1*. Phenotypic data suggested an increase in awn and root weights in inoculated transgenic plants. This triggered us to investigate flowering genes in roots in response to B26. Numerous flowering genes are identified in roots but were solely studied in the shoots. Bouché et al*.*^22^ reported that flowering genes in the roots of *Arabidopsis* are differentially expressed during flowering and concluded that roots may be involved in flowering by sending systemic signals or may participate actively in the regulation of flowering genes. However, the causal relationship was not very well established. In our study, the increase in expression of *FT1* in inoculated roots of *UBI:FT1* positively correlates with root weight. These transgenes are expressed under the control of maize ubiquitin constitutive promoter^17^ which upregulates the flowering gene expression, irrespective of bacterial treatment. Hence the increase in the transcript of *FT1* in inoculated roots of *UBI:FT1* is solely due to B26 inoculation. These results indicate that strain B26 modulates the transcription of flowering genes. This is the first report, according to our knowledge, that rhizobacteria can induce flowering genes in *B. distachyon* roots.

Non-symbiotic rhizobacteria contribute beneficial traits to colonized plants through bioactive compounds including, phytohormones^39^. These phytohormones influence the physiological processes of plants at very low levels^40^. Indeed, many studies demonstrated that rhizobacteria is associated with phytohormone concentrations and involved in homeostasis such as IAA, gibberellins, and IBA^41^. In our study, the endogenous phytohormones concentrations in the roots were modified by strain B26. Surprisingly, the concentrations of IAA and GA_7_ in inoculated Bd21-3 were lower than the control, but the transcripts of IAA were moderately up-regulated. This might be interpreted that strain B26 positively affected plant growth via metabolizing these phytohormones in the soil, a widespread trait among soil bacteria^42^. This plant hormonal homeostasis may rise from microbial consumption and production of hormones or fluctuations in plant hormones in planta^41^. Thus, plant-associated microbes can modulate plant metabolism by altering the plant hormone levels. Indeed, improved root growth of inoculated transgenic line *UBI:FT1* is attributed to GA_1_ production and in *UBI:VRN1* to GA_7_ and IAA. There is considerable evidence that gibberellins in grasses influence flower initiation^43^ Given that B26 affected endogenous amounts of phytohormones, the question then arises whether B26 effects on wild and transgenic lines resulted in larger root volume. We examined the roots of wild type and transgenic lines by Macro CT scanning that were inoculated with B26 and compared them to the control. Consistent with the induction of phytohormones in inoculated wild and transgenic lines, B26 had a positive effect on root volume of all accession lines. These results are congruent with preceding data and provide additional evidence of phytohormone modulation in *Brachypodium* roots by B26.

In summary, this report offers novel information about the long-term effects of a PGPR on plant development, advancing the knowledge on these relevant biological interactions. Our study shed new light on the involvement of strain B26 by influencing the flowering process in the roots. Key causal relationships cannot be established since we know little about the expression role of flowering genes in the *Brachypodium* roots and how they are connected to above-ground tissues. We also conclude that plant genotypes are critical to a successful interaction with PGPR.

## Methods

### Bacterial strain, growth, and inoculum preparation

The Plant Growth Promoting Rhizobacteria (PGPR) viz., *Bacillus velenzensis* strain B26^44^, formally known as *B. subtilis*^4^ was used in this study. The strain B26 was stored in 20% glycerol stocks in Lysogeny Broth (LB) (BDH chemical Ltd, Mississauga, ON, Canada) at − 80 °C. Revival of strain B26 was done on LB at 28 ± 1.0 °C on a rotatory shaker at 120 rpm until an OD_600_ of 1.0 (10^6^ CFU mL^−1^) was reached. Cells of strain B26 were centrifuged, washed, and suspended in a volume of phosphate buffer (1 M, pH 7) and used as inoculum for all experiments.

### Plant material and growth conditions of wild type and transgenic lines

Four *Brachypodium distachyon* accessions were selected based on their origins, vernalization requirements and flowering time. Selected accessions were Bd21, Bd21-3, Bd18-1 and Bd30-1 (Table S1). Wild type seeds were provided by Dr Jean-Benoit Charron, Macdonlad Campus, McGill University, Canada which were originally sourced from Dr David F. Garvin^45,46^, U.S Department of Agriculture (USDA)-Agriculture Research Service (ARS). 

*Growth conditions of wild-type B.distachyon accessions:* Seeds were sterilized following the methodology of Vain et al*.*^47^. Stratification and vernalization of seeds were done by placing them between two moist filter papers in a Petri dish and incubating them at 4 °C in the dark. The number of days for seed incubation was decided according to the vernalization requirement of wild type accessions (Table S1). After vernalization, seeds were sown in pots (6.35 × 6.35 × 7.62 cm) containing G2 Agro Mix® (Fafard et Frères Ltd, Saint-Remi, QC, Canada). Four sterile seeds were planted in each pot and pots were arranged in a Randomized Complete Block Design (RCBD). Pots were transferred to a growth cabinet (Conviron, Winnipeg, MB, Canada) with the light intensity of 150 μmoles m^2^ s^−1^, 16 h light and 8 h dark at day/night temperatures of 25 °C /23 °C. Every two weeks, plants were fertilized with 2 g/litre of N-P-K Fertilizer 20-20-20 (Plant Products Co. Ltd, Laval, QC, Canada).

#### Growth conditions of transgenic lines

Transgenic lines *UBI:FT1* and *UBI:VRN1* were used along with wild type Bd21-3. *UBI:FT1* encodes a phosphatidylethanolamine binding protein known as florigen that travels from leaves to the shoot apical meristem to induce flowering^17^.While *UBI:VRN1* encodes for floral homeotic MADS-box transcription factor^17^. Seeds of transgenic lines overexpressing flowering genes were kindly provided by Dr Daniel P Woods, University of California-Davis, U.S. Seeds were imported with approved import permit P-2019–01,394 from Canada Food Inspection Agency (CFIA). Seeds were sterilized as previously described for wild accession lines. Transgenic lines did not require vernalization, while the wild type was vernalized for three weeks at 4 °C in the dark. Four sterile seeds were planted in each pot and pots were arranged in a Randomized Complete Block Design (RCBD). Plants were grown in a controlled growth chamber with a higher light intensity of 300 μmoles m^2^ s^−1^, 20 h light and 4 h dark at day/night temperatures of 21 °C /18 °C as recommended^48^.

### Genotyping of Transgenic lines

To confirm the homozygosity of transgenic lines, PCR-based genotyping was carried out. DNA was extracted from young leaves of transgenic plants following the modified CTAB method. cDNA specific forward primer and pANIC vector AcV5 tag reverse primer were used to detect transgene (Table 3). Wild type Bd21-3 was used as control. The presence and absence of amplification confirmed the transgene. Single-band amplification was considered a homozygous plant containing transgene. Only homozygous plants were used.

**Table 3:** List of primers used in this study.

Gene of interest	Primer name	Primer sequence (5′ to 3′)	Product size (bp)	References
**Primers for B26 quantification**				
B26 Quantification	B26-F	CAAGTGCCGTTCAAATAG	565	^3^
	B26-R	CTCTAGGATTGTCAGAGG		
**Primer for transgene detection**				
pANIC vector AcV5 tag	BdAcV5-R	agaccagccgctcgcatctttccaag	100	^17^
**qRT-PCR primers for flowering genes**				
*FT1*	BdFT1-F	TTCGGGAACAGGAACGTGTCCAAC	100	^17^
	BdFT1-R	AGCATCTGGGTCTACCATCACGAG		
*FT2*	BdFT2-F	AGTACTTGCACTGGCTGGTCAC	115	
	BdFT2-R	CCGAGCTGCTGGAATAGAAGGAAC		
*VRN1*	BdVRN1_F	GCTCTGCAGAAGGAACTTGTGG	140	
	BdVRN1_R	CTAGTTTGCGGGTGTGTTTGCTC		
*VRN2*	BdVRN2_F	ATGCATGAGAGAGAGGCGAAGG	150	
	BdVRN2_R	TCGTAGCGGATCTGCTTCTCGTAG		
*Ubiquitin*	UBC18-F	GGAGGCACCTCAGGTCATTT	100	^30^
	UBC18-R	ATAGCGGTCATTGTCTTGCG		
**qRT-PCR primers for genes related to phytohormones**				
*Anthranilate synthase alpha subunit1(ASA1)*	ASA1-F	GCTCCAAGCCACAACACTAT	139 bp	BdiBd21-3.1G0905900.1
	ASA1-R	CCGCCTTATTCTCGCATTCT		
*Auxin responsive protein (IAA18)*	IAA18-F	AAGCCGTCACCTCAATCATC	119 bp	BdiBd21-3.2G0073500.1
	IAA18-R	TTCACGAACACGCCCTTT		
*Gibberellin 20-oxidase*	GA20ox1-F	AAGTCGCTGGCTTTCTTCC	105 bp	BdiBd21-3.1G0010900.1
	GA20ox1-R	CCACGTGAAATCCGGGTAAA		
DELLA PROTEIN	DELLA-F	CGTCAACTCAGTCTTCGAGAT	136 bp	BdiBd21-3.1G0148400.1
	DELLA-R	TGAGCCAGAGTTGTGGTTAG		

### B26 Inoculation and Assessment of Plant Growth Parameters of Wild type Accessions and Transgenic Lines

#### Experiment 1

To examine the differential response of *B. distachyon* to B26 inoculation, wild accession lines were inoculated with strain B26 at defined phenological growth stages using BBCH numerical scale^49^. Twenty-one days old plants (BBCH 23) were inoculated with 10 mL of B26 cells suspended in phosphate buffer (10^6^ CFU mL^−1^), while control plants received 10 mL of phosphate buffer per pot. Plants were harvested after 14 and 28 days post-inoculation (dpi) at defined phenological (BBCH 61) and (BBCH73) growth stages, respectively, and various phenotypic parameters were recorded. Five pots were harvested at each harvesting time point by carefully removing the substrate and washing the roots carefully. Growth parameters including Plant height, number of leaves, awns, tillers, fresh root and shoot weight were recorded. At each harvesting stage leaf and root samples were collected and stored at − 80 °C for downstream applications. The experiment was repeated twice.

#### Experiment 2

To determine the effect of inoculation on *B. distachyon* flowering, overexpressing transgenic lines were observed for plant growth parameters. 14-days old (BBCH 13) transgenic lines and wild type Bd21-3 were inoculated with 10 mL of B26 inoculum as described in the previous section. Data was recorded after 14 dpi (BBCH53), 28 dpi (BBCH69) and 42 dpi (BBCH87). At each harvesting time point, data of 5 pots per accession were recorded for plant height, number of leaves, awns, tillers, awn weight, fresh root and shoot weight. At each harvesting stage leaf and root samples were collected and stored at − 80 °C for downstream applications.

#### Experiment 3

To compare the total root volume between control and inoculated plants, macro CT-Scanning was done. A Semi-hydroponics system was developed for scanning of roots using Magenta GA-7 tissue culture boxes that were filled with sterile glass low alkali beads (Ceroglass, USA) saturated with Hoagland’s solution as fully described in Sharma et al*.*^5^. Pre-germinated seeds of wild type Bd21-3, transgenic lines *UBI:FT1* and *UBI:VRN1* (6 seeds/box) were transferred to Magenta boxes where each box is an experimental unit. Boxes were incubated in a controlled growth cabinet (Conviron, Canada) with light intensity of 300 μmoles m^2^/s,16 h light and 8 h dark at day/night temperatures of 21 °C/18 °C. After 14 days of growth, three boxes of each line received B26 inoculum (500 µL OD_600_ of 1) suspended in phosphate buffer (1 M, pH), and three control boxes received 500 μL of phosphate buffer alone. All boxes were incubated in a controlled growth cabinet. A total of 6 Magenta boxes were used per line.

### B26 quantification in root and leaves of selected wild type *B. distachyon* accessions

Quantification of B26 DNA copy number was performed in roots and leaves of Bd21-3 and Bd30-1 at 14, and 28 dpi using qPCR. Genomic DNA was extracted from 1 g of powdered tissue using the modified CTAB method. DNA from the pure culture of B26 was also extracted from a single B26 colony using the boiling method^50^. For detection purposes, conventional PCR was done using B26 strain-specific primers in inoculated leaves and roots of selected accessions. B26 bacterial DNA served as a positive control in PCR. Cloning and qPCR reactions were performed as described in Gagne-Bourque et al*.*^3^. To calculate the quantity of bacterial DNA in inoculated roots and leaves, Cq (Cycle quantification) values of plant DNA were correlated with Cq values in the standard curve. Moreover, for reliability of the designed method, correlation coefficient and the amplification efficiency were calculated from the formula X_o_ = E_AMP_^(b-Cq)^ = 10^(Cq-b)/m))^, where X_o_ = initial reaction copies, E_AMP_ = Exponential amplification, b = y-intercept of the standard curve (log_10_ of copies), m = slope of standard curve.

### Phytohormone analysis

To determine the effect of inoculation on phytohormones, endogenous levels of plant phytohormones including auxin, cytokinin, gibberellins and abscisic acid was measured using the modified protocol of Li et al*.*^51^. Inoculated and control roots of Bd21-3, transgenic lines; *UBI:FT1* and *UBI:VRNI1* from Experiment 2 were subjected to phytohormone analysis after 28dpi. Root samples were crushed in liquid nitrogen. Samples were sent in triplicates to The Metabolomics Innovation Centre, UVic-Genome BC Proteomics Centre, Victoria, BC, Canada. Briefly, 100 mg of each sample was precisely weighed into a 2-mL safe-lock Eppendorf tube. 4 µL of 5% formic acid in water per mg of raw tissue and two 4-mm stainless steel balls were added. The sample was homogenized at a shaking frequency of 30 Hz on a MM 4000 mixer mill for 1 min three times. Methanol, at 16 µL per mg raw tissue was then added. The sample was homogenized again for 1 min three times, followed by sonication in an ice-water bath for 5 min and centrifugal clarification at 21,000*g* and 10 °C for 10 min. The clear supernatant was collected for the analysis of auxins, cytokinin, gibberellins and abscisic acid. Phytohormones were analysed with UPLC- multiple-reaction monitoring (MRM) mass spectrometry on an Agilent 1290 UHPLC coupled to an Agilent 6495B QQQ mass spectrometer equipped with an ESI source which was operated in the negative-ion mode. LC separation was carried out on a C18 UPLC column (2.1 × 150 mm, 1.8 µm). Concentrations of the detected compounds in the sample solutions were calculated by interpolating the constructed linear-regression calibration curve with the measured analyte-to-internal standard peak area ratios.

### CT scanning of wild type Bd21-3 and transgenic lines

The total root volume of inoculated and non-inoculated wild accession Bd21-3, transgenic lines *UBI:FT1* and *UBI:VRN1* grown in magenta boxes were compared by performing macro CT- scanning at 28 dpi. The root systems were scanned using macro-CT scanning with the Canon CT Aquilion Prime SP at the CT Scanning Laboratory for Agricultural and Environmental Research, Macdonald Campus of McGill University, Sainte-Anne-de-Bellevue, Quebec, Canada. Each magenta box served as one replicate with six plants per experimental treatment. Each box was in a standing up position at the time of CT scanning, and the lower part of each box was CT scanned individually. The main CT scanning settings were: tube voltage, 80 kV; tube current, 50 mA; voxel dimensions, 0.188 × 0.188 × 0.5 mm^3^ (X × Y × Z, with Z the axis of the CT scanner couch). Given the presence of glass beads (between which roots grew), root amount (instead of root system architecture) was studied and estimated from the CT scanning data, more particularly from the histogram of CT numbers. A CT number (CTN) is an indirect measure of density; a macro-CT scanner is calibrated so that air CTN = − 1000, water CTN = 0, CTN for glass beads appeared to be around + 2000. Because of edge effects, the non-flat surface of the growth medium and the variable filling with glass beads, root amount was estimated within two volumes, a “larger volume V” and a “smaller volume V”. Size of volumes (in voxels): larger volume V, 100 × 300 × 100 (53,016 mm^3^); smaller volume V, 100 × 150 × 80 (21,206 mm^3^). For comparison purposes, two ranges of CT numbers were used in root amount estimation with the smaller volume V (to define so-called pseudo-root voxels): [− 700, + 300] and [− 800, + 400]. Only the range [− 700, + 300] was used with the largest volume.

### RNA extraction, cDNA synthesis and qRT-PCR analysis

#### Transcript abundance of flowering genes in selected B. distachyon wild type and transgenic lines

In response to B26 inoculation, we decided to choose the best phenotypic performer in terms of growth parameters (Bd21-3) and the least phenotypic performer (Bd30-1). We examined the gene expression of *Brachypodium* flowering pathway genes viz., *FT1*, *FT2*, *VRN1* and *VRN2* in leaves of Bd21-3 and Bd30-1 from Experiment 1 at 14 dpi and 28 dpi. To study the genotypic response of B26 on *B. distachyon* transgenic lines, transcript abundance of *FT1* and *VRN1* was measured in control and inoculated transgenic lines; *UBI:FT1* and *UBI:VRN1* along with wild type Bd21-3 roots and leaves from Experiment 2 at 28dpi. Briefly, total RNA was extracted from flash-frozen pulverized 100 mg of inoculated and control tissues using Spectrum™ Plant Total RNA Kit (Sigma Aldrich, US) following the manufacturer’s protocols. One Script RT ABM kit (Vancouver, Canada) was used for reverse-transcription of RNA (500 ng) following the manufacturer’s protocols. PCR assays were performed on three biological replicates and two technical replicates. Primer details are present in Table 3. The conditions for qRT-PCR were adjusted for each primer set. PCR amplification was performed in a 10 µL reaction following the protocol of Sharma et al.^5^. The 2^−ΔΔ*C*T^ method^52^ was applied to normalize the target gene over the housekeeping genes *UBC18*. Bestkeeper tool was used to compare housekeeping genes *UBC18* and *ACTIN2*. *UBC18* had the lowest coefficient variation as compared to *ACTIN2* so *UBC18* was chosen for the normalization*.*

#### Transcript abundance of genes encoding phytohormones in Bd21-3

The effect of B26 inoculation on the phytohormone production by *B.distachyon* roots was quantified using qRT-PCR. Transcript abundance of auxin and gibberellins biosynthesis genes was measured only in roots of Bd21-3 from Experiment 2 at 28 dpi. Primer sets (Table 3) were designed based on gene sequences retrieved from Phytozome Bd21-3 v1.1 genome (Phytozome v12.1, https://phytozome.jgi.doe.gov/pz/portal.htmL. Primers were designed online from IDT website using Primer Quest Tool (https://www.idtdna.com/PrimerQuest/Home/Index). To confirm the specificity of Primers, sequences were checked for hairpins and hetero-dimer formations using the Oligoanalyzer tool (http://www.idtdna.com/calc/analyzer) and submitted to Nucleotide Blast at NCBI (http://www.ncbi.nlm.nih.gov/) and were custom synthesized by Integrated DNA Technologies (IDT, Iowa, USA). One hundred milligrams of tissue was subjected to RNA extraction. cDNA preparation and qRT-PCR were performed as described in the previous section.

### Statistical analysis

Data of all experiments were analysed using IBM Statistics SPSS Version 24(SPSS Inc., Chicago, IL). Comparison of means was performed by independent student t-test for comparison between control and inoculated samples. Tukey’s test was performed to compare the means of multiple treatments. We considered a *p* < 0.05 acceptable for statistical significance. Experiments 1 and 2 were performed using 5 replicates for each control and inoculated pots. To prevent contamination of treatments, two growth chambers were used for control and inoculated plants. To study the confounding effect of growth chambers, the experiments were repeated twice by exchanging the growth chambers of treatment with control plants.

## Supplementary Information

Supplementary Information.
