# Supplementary material for: Bacillus velezensis strain B26 modulates the inflorescence and root architecture of Brachypodium distachyon via hormone homeostasis
Source: Sci Rep. 2022 May 13;12:7951. doi: 10.1038/s41598-022-12026-6 (PMC9106653; doi:10.1038/s41598-022-12026-6)
Supplement: Supplementary file 1 — Supplementary Information. [file 41598_2022_12026_MOESM1_ESM.pdf]

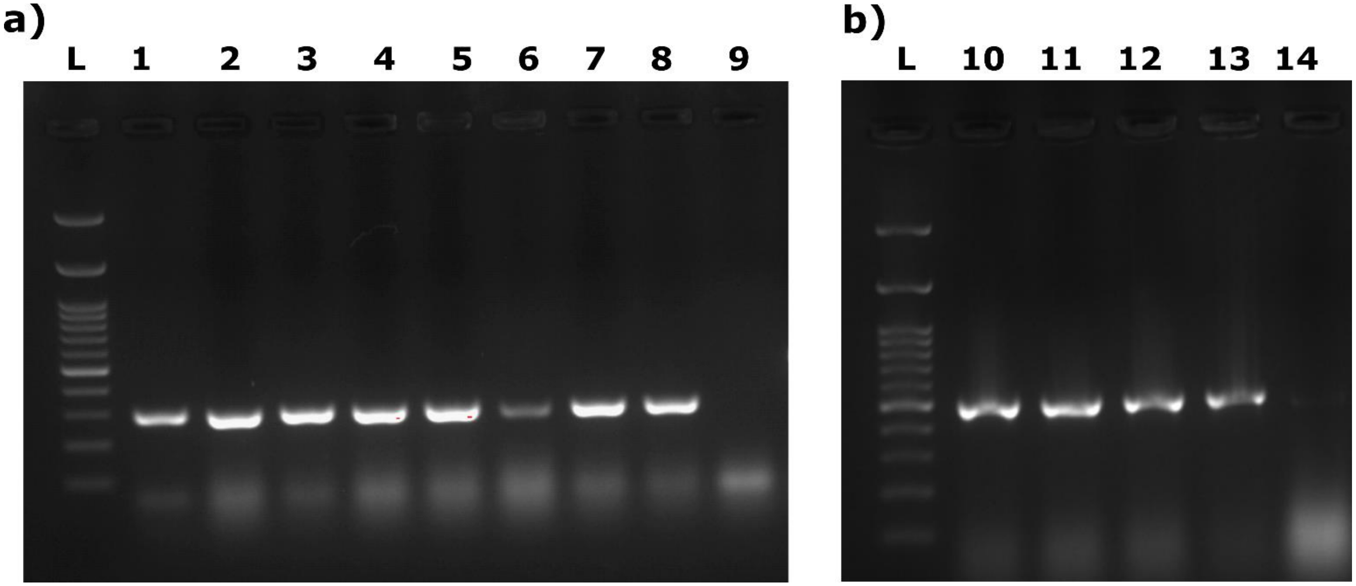

Supplementary Figure S1. Genotyping of flowering transgenic line a) *UBI:VRN1* and b) *UBI:FT1* using AcV5-R tag and gene-specific forward primer.
L=100bp DNA ladder, Lane 1-8: Amplification of *UBI:VRN1* DNA; Lane 10-13: Amplification of *UBI:FT1* DNA; Lane 9 and 14: Bd21-3 DNA. Gel picture is complete only empty wells were cropped.

Supplementary Table S1: *Brachypodium distachyon* genotypes used in this study

**PI accession**	**Geographic origin**	**Growth Habitat**	**Vernalization Requirement**	**Flowering Class**
Bd21	Iraq	Spring	2-3 weeks	Extremely Rapid Flowering
Bd21-3	Iraq	Spring	2-3 weeks	Rapid Flowering
Bd30-1	Spain	Spring	2-3 weeks	Intermediate Rapid Flowering
Bd18-1	Turkey	Winter	4-5 weeks	Delayed Flowering

Supplementary Table S2: Growth Response of *B. distachyon* accessions lines in response to *B.velezensis* strain B26 inoculation

			Growth Parameters[$]					
**Time Point**	**Accessions**	**Treatment**	**Plant Height(cm)**	**No. of leaves**	**No. of Tillers**	**No. of awns**	**Root Weight (g)**	**Shoot Weight (g)**
**14dpi[&]**		B+	11.85±1.00^{a}	14.50±2.46^{a}	3.60±0.29^{a}	3.00±0.54^{a}	0.41±0.10^{a}	0.98±0.11^{a}
	**Bd21**	B-	11.60±1.75^{a}	14.60±3.48^{a}	5.40±1.12^{a}	1.20±0.20^{b}	0.27±0.08^{a}	0.28±0.07^{b}
		%increase†	2.15%	-0.68%	-33.33%	**150%***	51.85%	**250%***
		B+	17.35±0.64^{a}	19.00±2.51^{a}	4.60±0.60^{a}	2.40±0.74^{a}	0.68±0.13^{a}	1.48±0.11^{b}
	**Bd21-3**	B-	16.00±1.44^{a}	21.30±3.68^{a}	4.90±1.05^{a}	1.40±0.40^{a}	0.65±0.13^{a}	0.78±0.11^{a}
		%increase	8.09%	-2.30%	-6.31%	71%	4.61%	**89.71%***
		B+	15.23±0.56^{a}	18.00±1.43^{b}	4.60±0.29^{a}	0	1.53±0.30^{a}	3.63±0.72^{a}
	**Bd18-1**	B-	11.35±1.40^{b}	10.10±2.17^{a}	3.20±0.68^{a}	0	0.73±0.14^{a}	2.24±0.44^{a}
		%increase	**34.18%***	**78.21%***	43.75%	NA	109.58%	62.05%
		B+	14.80±2.40^{a}	9.45±0.73^{a}	2.40±0.48^{a}	3.50±0.97^{a}	0.11±0.02^{a}	0.34±0.10^{a}
	**Bd30**	B-	12.25±0.79^{a}	9.20±0.51^{a}	2.80±0.20^{a}	1.90±0.10^{a}	0.13±0.02^{a}	0.27±0.01^{a}
		%increase	20.81%	2.71%	-14.28%	84.21%	-15.38%	25.92%
**28dpi**	**Bd21**	B+	12.37±1.34^{a}	29.80±4.45^{a}	4.30±0.94^{a}	3.80±0.73^{a}	0.85±0.12^{a}	1.62±0.18^{a}
		B-	11.90±1.80^{a}	21.60±3.82^{a}	4.40±0.69^{a}	1.40±0.40^{b}	0.62±0.18^{a}	0.86±0.34^{a}
		%increase†	3.97%	37.96%	-2.22%	**171%***	37.09%	88.37%
		B+	20.70±1.00^{a}	41.50±3.88^{a}	7.10±0.55^{a}	4.20±0.80^{a}	1.36±0.21^{a}	3.72±0.56^{a}
	**Bd21-3**	B-	13.35±1.72^{b}	19.60±4.77^{a}	4.90±1.06^{b}	1.80±0.20^{b}	0.62±0.13^{b}	1.93±0.42^{b}
		%increase	**55.05%***	**111.73%***	**44.89%***	**133%***	**119.35%***	**92.74%***
		B+	18.25±1.11^{a}	30.30±5.00^{a}	6.20±1.04^{a}	0	1.00±0.18^{a}	0.65±0.07^{a}
	**Bd18-1**	B-	16.05±2.53^{a}	25.50±4.83^{a}	5.90±1.10^{a}	0	1.06±0.05^{a}	0.62±0.06^{a}
		%increase	13.70%	18.82%	5.08%	NA	-5.66%	4.83%
		B+	14.95±0.82^{a}	7.80±1.03^{a}	2.30±0.48^{a}	3.70±0.64^{a}	0.17±0.03^{a}	0.65±0.07^{a}
	**Bd30-1**	B-	13.25±0.68^{a}	8.40±1.40^{a}	2.00±0.20^{a}	3.50±0.67^{a}	0.24±0.03^{a}	0.62±0.06^{a}
		%increase	12.83%	-7.14%	15.00%	5.71%	-29.16%	3.22%

&, days post-inoculation

$, Growth parameters represent the average of 5 pots per treatment or 10 plants ± standard error

†, percentage increase relative to the control treatments

*, statistically significant values within a row between control and treatment at $p < 0.05$ according to Independent Student t-test

NA, Not Applicable

Supplementary Table S3: ***Brachypodium*** **growth parameters of transgenic lines and wild type inoculated with strain B26**

			Growth Parameters$						
	**Accessions**	**Treatment**	**Plant Height(cm)**	**No. of leaves**	**No. of Tillers**	**No. of awns**	**Root weight (g)**	**Shoot Weight (g)**	**Awn weight(g)**
**14dpi&**	**Bd21-3**	B-	16.77 ± 0.84a	9.47 ± 0.80a	2.33 ± 0.18a	1.27 ± 0.65a	0.19 ± 0.04a	0.66 ± 0.10a	0.05 ± 0.03a
		B+	19.27 ± 0.85a	10.27 ± 0.48a	2.60 ± 0.19a	2.33 ± 1.08a	0.20 ± 0.05a	0.78 ± 0.09a	0.07 ± 0.03a
		%increase†	**14.91**	**8.45**	**11.43**	**84.21**	**2.39**	**18.88**	**36.19**
	UBI:FT1	B-	18.07 ± 0.21a	7.47 ± 0.49a	2.27 ± 0.16a	3.00 ± 0.41a	0.11 ± 0.02b	0.826 ± 0.06a	0.19 ± 0.03a
		B+	18.30 ± 1.06a	7.00 ± 1.13a	2.20 ± 0.13a	3.27 ± 0.29a	0.18 ± 0.03a	0.834 ± 0.14a	0.20 ± 0.01a
		%increase	**1.29**	**-6.25**	**-2.94**	**8.89**	**57.89***	**0.97**	**3.31**
	UBI:VRN1	B-	17.10 ± 0.12a	11.47 ± 0.23a	3.80 ± 0.29a		0.33 ± 0.02a	1.40 ± 0.07a	NA
		B+	17.23 ± 0.15a	12.13 ± 0.23a	4.07 ± 0.07a		0.38 ± 0.05a	1.42 ± 0.05a	NA
		%increase	**0.78**	**5.81**	**7.02**	**NA**	**15.15**	**1.29**	**NA**
**28dpi**	**Bd21-3**	B-	25.20 ± 1.10b	13.47 ± 1.06a	2.40 ± 0.16a	4.53 ± 0.47b	0.12 ± 0.01b	1.15 ± 0.09a	0.51 ± 0.04b
		B+	29.40 ± 0.69a	9.93 ± 1.34a	2.07 ± 0.27a	6.07 ± 0.42a	0.78 ± 0.09a	1.64 ± 0.24a	0.72 ± 0.07a
		%increase†	**16.66***	**-26.24**	**-13.89**	**34***	**551.67***	**42.96**	**42.52***
	UBI:FT1	B-	21.77 ± 0.39a	7.73 ± 0.24a	2.13 ± 0.08a	4.73 ± 0.46 a	0.18 ± 0.06b	0.66 ± 0.21b	0.66 ± 0.09a
		B+	23.73 ± 0.85a	8.87 ± 1.07a	2.33 ± 0.21a	4.87 ± 0.54a	0.42 ± 0.08a	1.78 ± 0.12a	0.82 ± 0.10a
		%increase	**9.04**	**14.66**	**9.37**	**2.89**	**132.38***	**161.97***	**25.73**
	UBI:VRN1	B-	23.70 ± 0.40b	23.33 ± 1.22a	4.60 ± 0.24a		2.00 ± 0.25a	3.84 ± 0.14a	NA
		B+	25.32 ± 0.55a	22.73 ± 0.71a	4.00 ± 0.00b		1.97 ± 0.31a	2.44 ± 0.37b	NA
		%increase	**6.83***	**-2.57**	**-13.04***	**NA**	**-1.70**	**-36.52***	**NA**
**42dpi**	**Bd21-3**	B-	28.00 ± 1.69a	9.08 ± 0.98a	2.07 ± 0.07a	7.92 ± 1.06a	0.25 ± 0.15b	1.77 ± 0.05a	0.81 ± 0.03 a
		B+	28.42 ± 0.60a	10.83 ± 1.55a	2.25 ± 0.25a	8.33 ± 0.49a	0.65 ± 0.15a	2.18 ± 0.51a	1.00 ± 0.10a
		%increase†	**1.49**	**19.31**	**8.87**	**5.18**	**160***	**23.35**	**23.46**
	UBI:FT1	B-	23.00 ± 0.93a	8.75 ±1.40a	2.08 ± 0.08a	7.17 ± 0.29a	1.04 ± 0.21b	2.67 ± 0.40a	0.99 ± 0.08a
		B+	22.33 ± 0.89a	6.75 ± 0.55a	2.67 ± 0.47a	9.33 ± 1.16a	2.86 ± 0.35a	3.22 ± 0.23a	1.13 ± 0.15a
		%increase	**-2.90**	**-22.86**	**28.00**	**30.23**	**175.88***	**20.60**	**14.14**

**42dpi**		B-	27.17 ± 0.74a	25.83 ± 1.37a	5.08 ±0.21a	12.00 ± 3.84a	1.93 ± 0.45b	3.15 ± 0.65a	0.64 ± 0.12a
	UBI:VRN1	B+	27.67 ± 3.11a	25.88 ± 4.21a	4.33 ± 0.19a	10.17 ± 1.43a	2.75 ± 0.83a	3.27 ± 0.60a	0.67 ± 0.23a
		%increase	**1.84**	**0.16**	**-14.75**	**-15.28**	**42.46***	**3.73**	**-4.48**

&, days post inoculation

$, Growth parameters represent the average of 5 pots per treatment or 10 plants ± standard error

†, percentage increase relative to the control treatments

*, statistically significant values within a row between control and treatment at $p < 0.05$ according to Independent Student t-test

NA, Not Applicable
